# HSP70 Expression Signature in Renal Cell Carcinoma: A Clinical and Bioinformatic Analysis Approach

**DOI:** 10.3390/genes14020355

**Published:** 2023-01-30

**Authors:** Noha M. Abd El-Fadeal, Alia Ellawindy, Mohammed A. Jeraiby, Safaa Y. Qusti, Eida M. Alshammari, Ahmad Khuzaim Alzahrani, Ezzat A. Ismail, Ziad Ehab, Eman A. Toraih, Manal S. Fawzy, Marwa Hussein Mohamed

**Affiliations:** 1 Department of Medical Biochemistry and Molecular Biology, Faculty of Medicine, Suez Canal University, Ismailia 41522, Egypt; 2 Center of Excellence in Molecular and Cellular Medicine, Faculty of Medicine, Suez Canal University, Ismailia 41522, Egypt; 3 Oncology Diagnostic Unit, Faculty of Medicine, Suez Canal University, Ismailia 41522, Egypt; 4 Medical Genetics Unit, Department of Histology and Cell Biology, Suez Canal University, Ismailia 41522, Egypt; 5 Department of Biochemistry, Faculty of Medicine, Jazan University, Jazan 82621, Saudi Arabia; 6 Department of Biochemistry, Faculty of Science, King Abdulaziz University, Jeddah 21589, Saudi Arabia; 7 Department of Chemistry, College of Sciences, University of Ha’il, Ha’il 2440, Saudi Arabia; 8 Medical Laboratory Technology, Faculty of Applied Medical Sciences, Northern Border University, Arar 91431, Saudi Arabia; 9 Department of Urology, Faculty of Medicine, Suez Canal University, Ismailia 41522, Egypt; 10 Faculty of Medicine, Mansoura University, Mansoura 21955, Egypt; 11 Division of Endocrine and Oncologic Surgery, Department of Surgery, Tulane University School of Medicine, New Orleans, LA 70112, USA; 12 Department of Biochemistry, Faculty of Medicine, Northern Border University, Arar 1321, Saudi Arabia

**Keywords:** gene expression, HSP70, HSPA4, in silico analysis, prognosis, RCC, survival, qRT-PCR

## Abstract

Heat shock proteins (HSPs) are cytoprotective against stressful conditions, as in the case of cancer cell metabolism. Scientists proposed that HSP70 might be implicated in increased cancer cell survival. This study aimed to investigate the *HSP70* (*HSPA4*) gene expression signature in patients with renal cell carcinoma (RCC) in correlation to cancer subtype, stage, grade, and recurrence, combining both clinicopathological and in silico analysis approaches. One hundred and thirty archived formalin-fixed paraffin-embedded samples, including 65 RCC tissue specimens and their paired non-cancerous tissues, were included in the study. Total RNA was extracted from each sample and analyzed using TaqMan quantitative Real-Time Polymerase Chain Reaction. Correlation and validation to the available clinicopathological data and results were executed. Upregulated *HSP70* (*HSPA4*) gene expression was evident in RCC compared to non-cancer tissues in the studied cohort and was validated by in silico analysis. Furthermore, *HSP70* expression levels showed significant positive correlations with cancer size, grade, and capsule infiltration, as well as recurrence in RCC patients. The expression levels negatively correlated with the overall survival (*r* = −0.87, *p* < 0.001). Kaplan–Meier curves showed lower survival rates in high HSP70 expressor group compared to the low expressors. In conclusion, the HSP70 expression levels are associated with poor RCC prognosis in terms of advanced grade, capsule infiltration, recurrence, and short survival.

## 1. Introduction

Renal cell carcinoma (RCC), arising from renal tubular epithelial cells, is one of the most lethal kidney cancers [1]. The incidence of RCC has increased worldwide, and accounts for 2.2% of cancer-diagnosed cases and 1.8% of deaths [2,3]. Regretfully, RCC is usually diagnosed incidentally during imaging for other conditions, making timely intervention not possible [4]. RCC comprises several histopathological subtypes including clear cell carcinoma (representing up to 75% of cases), papillary carcinoma, chromophobe carcinoma, and other rare subtypes [5]. Both environmental (e.g., smoking, obesity, hypertension, chronic use of pain medications) and genetic factors have been implicated in the risk of RCC [6]. Identifying molecular players in RCC pathogenesis gives hope for earlier identification and possible treatment of the disease.

Heat shock proteins (HSPs) are a family of proteins that aid in properly folding of proteins and destroying misfolded or unfolded proteins [7]. Their molecular weights range from 15 to 90 kDa and are classed accordingly. Using a process known as molecular chaperoning, HSPs are exploited as cytoprotective against stress conditions in cancer cells to maintain homeostasis [5,6]. Furthermore, HSPs have been shown to participate in immunogenicity and immune recognition in different cancer types [8,9]. This is owing to their ability to pick up the spontaneously released HSP-peptide complexes formed by antigen-presenting cells from dying tumor cells and their ability to facilitate cancer antigen detection by cytotoxic T-cell activity [10].

HSP70 has been shown to play a crucial role in cancer [11]. HSP70 is highly expressed on the surface of tumor cells [12] which actively release HSP70 surface-positive exosomes, stimulating natural killer (NK) cells [13]. Moreover, apoptotic cancer cells liberate HSP70, which serves as damage-associated molecular patterns (DAMPs) [14,15]. DAMP has a strong immunogenic potential which elicits a robust antitumor T-cell response [16,17]. Long-term exposure of immune cells to free HSP70 after radiation, on the other hand, has been demonstrated to promote immunological tolerance and accelerate tumor progression [18]. Furthermore, a small dose of the HSP70-peptide complex is sufficient to activate antitumor immunity [19]. When applied to endogenous HSP70 released by tumor cells, it is possible to hypothesize that early HSP70 release acts as a tumor suppressor; nevertheless, HSP70 overproduction causes tumor progression [20]. 

HSP70 has been reported to be overexpressed in malignant tumors and may serve as a biomarker for poor prognosis [20]. However, data on the significance of HSP70 expression in RCC is limited and remains controversial [21,22,23,24]. Therefore, the current study aimed to explore HSP70 gene expression in RCC in Egyptian patients and to evaluate how it relates to RCC features and prognosis. A bioinformatic analysis of HSP70 and its role in RCC was also performed. 

## 2. Materials and Methods

### 2.1. Study Population

This case-control study involved 65 paired archived formalin-fixed paraffin-embedded (FFPE) samples collected from patients with RCC subjected to surgical treatment by partial or radical nephrectomy from October 2015 to December 2019 at the Suez Canal University Hospitals and referred cases to Oncology Diagnostic Unit, Ismailia, Egypt. Patients’ medical data were obtained from their records archive, and any missing data was taken by interviewing the patient (if available). We excluded patients who took chemotherapy or radiotherapy before the surgical resection of the tumor to eliminate bias and confounders. Also, patients with incomplete clinical and/or histopathological data were excluded. The research was carried out in accordance with the Helsinki Declaration’s ethical and legal standards. Ethical approval was taken from the Suez Canal University Faculty of Medicine Ethical Committee (approval code 5054#, 4 October 2022). Patient consent was waived as the study was carried out retrospectively on archived samples.

### 2.2. Histopathology Assessment

Tumor grading and staging were performed by staining the RCC tissues (sections of 4 μm in thickness) using hematoxylin and eosin [25,26]. Other sections (5 μm in thickness) were collected on sterilized (ribonuclease-free) Eppendorf for genetic studies. The histopathological assessment was blindly performed according to the “International Society of Urological Pathology (ISUP) Vancouver Modification of WHO, Histologic Classification of Kidney Tumors” [27]. Grading and staging were done according to international standard protocols [28,29].

### 2.3. Gene Expression Analysis

The FFPE sections were incubated with proteinase K for 15 min to digest the cellular proteins; then samples were subjected to second incubation at 80 °C for 15 min to remove the nucleic acid cross-links and allow for better RNA yield. Samples were treated for 2 h at 37 °C using RNase -free DNase 1 to remove any DNA contamination, followed by extraction of total RNA using the protocol of the RNeasy FFPE kit (Cat#74404, Qiagen, Hilden, Germany). The extracted RNA was evaluated for quality and quantity using gel electrophoresis and a Nanodrop-1000 spectrophotometer (Nanodrop Technologies, Wilmington, DE, USA). Then it was subjected to reverse transcription using high-capacity cDNA reverse transcriptase kits (Cat#4374966, Applied Biosystems, Waltham, MA, USA) on Veriti™ 96-Well Fast Thermal Cycler (Cat#4375305, Applied Biosystems, Waltham, MA, USA). The prepared master mix for each run followed in RT-PCR was detailed in previous work [30]. The RT program was carried out at “25 °C for 10 min, followed by 37 °C for 120 min, and finally 85 °C for 5 min, then hold at 4 °C”. Finally, the *HSP70* mRNA expression level was quantified compared to glyceraldehyde 3-phosphate dehydrogenase (*GAPDH*) as a housekeeping gene following the protocol described previously [31], using qRT-PCR on StepOne Plus™ Real-Time PCR System (Cat#4376600, Applied Biosystems, Waltham, MA, USA) using TaqMan assays (ID: Hs00382884_m1 for *HSP70* and Hs02786624_g1 for *GAPDH*) and appropriate negative controls in each run. The program was set as follows: “initial 5 min at 95 °C, followed by denaturation for 15 s at 95 °C, annealing for 1 min at 60 °C, and elongation for 1 min at 72 °C, repeated 40 cycles”. Each sample was run in triplicate, and the average quantitative cycle (Cq; the cycle number at which the measured fluorescence during the Real-Time PCR passed a fixed threshold) value was assigned to calculate the fold change of the *HSP70* gene. All the qPCR runs were carried out in accordance with the “Minimum Information for Publication of Quantitative Real-Time PCR Experiments; MIQE” guidelines [32].

### 2.4. Gene Expression Data Analysis

The relative quantification of the investigated *HSP70* to *GAPDH* in patients samples compared to controls (non-cancerous samples) was calculated using the Livak equation that depends on: “2^−ΔΔCq^; where ΔΔCq = (Cq HSP − Cq GAPDH)_RCC_ − (Cq HSP70 − Cq GAPDH) _controls mean_” [33].

### 2.5. Bioinformatic Analysis

#### 2.5.1. HSP70 Structural and Functional Analysis

The in silico structural/functional analysis for the *HSP70* (*HSPA4*) gene and protein was screened in different databases such as the “National Center of Biotechnology Institute; NCBI (https://www.ncbi.nlm.nih.gov/gene/3308/), Ensemble (www.ensembl.org), gene cards (www.genecards.org), UniProt knowledge base (https://www.uniprot.org/), “ProMod3 3.2.1” for homology modeling (https://swissmodel.expasy.org), Compartments for subcellular localization (https://compartments.jensenlab.org/), “NetworkAnalyst 3.0” for transcriptional factors interaction and related microRNAs networks generation (https://www.networkanalyst.ca), String database version 11.5 for protein-protein association network with gene ontology and functional enrichment analysis (https://string-db.org/), and the “Gene Multiple Association Network Integration Algorithm; Gene Mania” version 3.6.0 (https://genemania.org). All databases were last accessed on 1 December 2022.

#### 2.5.2. In Silico Analysis Using Genotype-Tissue Expression (GTEx) and the Cancer Genome Atlas (TCGA) Tissues for Assessing the Expression of HSP70 in RCC

##### Gene Expression Profiling Interactive Analysis (GEPIA) Expression and Correlation Analysis

The GEPIA database (http://gepia.cancer-pku.cn/index.html) (accessed on 2 December 2022) was employed to compare the expression level of *HSP70* in tumors and normal tissues. The expression signature of the *HSP70* was compared in three different RCC subtypes, including kidney chromophobe, kidney renal clear cell carcinoma, and kidney renal papillary cell carcinoma. Also, the violin plot was applied to measure the expression in different tumor stages. Furthermore, GEPIA was used to calculate the correlations between *HSP70* expression and RCC overall survival and disease-free survival in the three subtypes.

##### MEXPRESS Gene and Clinical Data Analysis

The MEXPRESS database (https://mexpress.be) (last accessed 2 December 2022) was used for visualizing the association of HSP70 expression with the clinical data and copy number variations using TCGA samples [34].

##### cBioPortal Database Analysis

The cBioPortal database platform (https://www.cbioportal.org/datasets) (last accessed 2 December 2022) explored multidimensional cancer genomics data and the potential targeted pathway analysis.

### 2.6. Statistical Analysis

To estimate the total study sample size at power 80, “G*Power (version 3.0.10)” [35] was applied at medium effect size = 0.5 and α error probability = 0.05. The estimated sample size at the specified conditions was 64 for each subgroup (cancer vs. non-cancerous). Mann–Whitney U and Kruskal–Wallis tests were used. Spearman’s correlation test was employed, and the correlation coefficient was reported in the correlation matrix plot. The “receiver operating characteristic” (ROC) curve was generated to explore the utility of *HSP70* expression level for discriminating patients with RCC from controls. A Kaplan–Meier curve was generated for disease-free survival. The log-Rank test was used for analysis. A two-sided *p*-value < 0.05 was set to be significant. Analysis was performed using R Studio Build 544 and SPSS “version 27.0 (IBM Corp., Armonk, NY, USA)”.

## 3. Results

### 3.1. Baseline Characteristics of the Study Group

**Figure 1:**
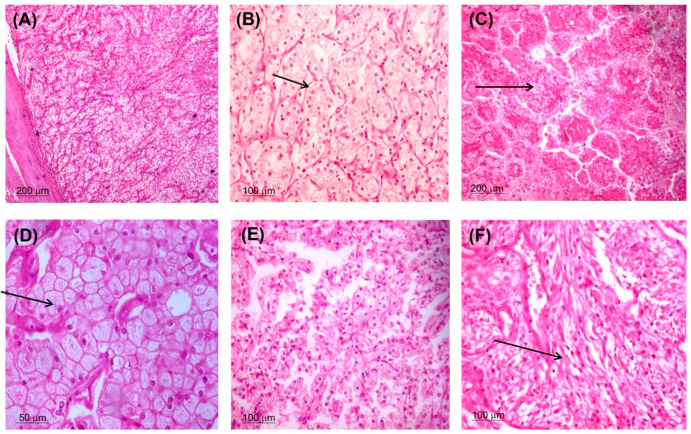
Morphological types of RCC. Clear cell type ((**A**) ×100 and (**B**) ×200) with clear cytoplasm, well defined cell borders, and nuclear grade 2 (black arrow). Papillary RCC ((**C**) ×100) showed papillary structures with core containing foam cells (black arrow) and covered by columnar cells. Chromophobe RCC ((**D**) ×400) formed of nests of cells with abundant eosinophilic cytoplasm and raisin nuclei (black arrow). RCC with variable features suspected to be unclassified type that need genetic study ((**E**) ×200). RCC with sarcomatoid features (black arrow) and considered grade 4 ((**F**) ×200).

Sixty-five RCC patients (43 females and 22 males) were included in the current study. The mean age was 52.4 ± 11.7 years (range: 20–79). The clinicopathological characteristics of the RCC patients are demonstrated in Table 1. Two-thirds of patients (67.7%) had cancer on the right side. Pathological analysis revealed 13 patients with advanced tumor size (T3/T4), and 25 cases presented with poor pathological grade (G3/G4). The most common type of cancer was kidney renal clear cell carcinoma (KIRC) (49%) followed by kidney papillary cell carcinoma (KIPC) (29.2%) (Figure 1). Evidence of capsular infiltration was observed in 40% of cases. After a follow-up of 18.1 ± 6.4 months (range: 4–29 months), 34 patients (52.3%) developed recurrence, and only 10.8% of cases survived.

### 3.2. HSP70 Gene Expression Analysis in Studied Patients with RCC

**Figure 2:**
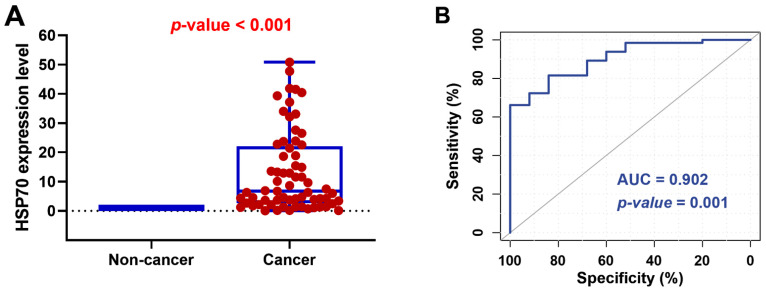
*HSP70* gene expression profile. (**A**) Expression in non-cancerous and cancerous renal tissue of renal cell carcinoma (RCC). Data are represented as medians. The box defines upper and lower quartiles (25% and 75%, resp.), and the error bars indicate upper and lower adjacent limits. Expression levels of HSP70 were normalized to GAPDH. Fold change was calculated using the delta-delta CT method (2^−ΔΔCT^) compared to normal renal tissues. The median and quartile values of patients are demonstrated in blue. The dashed line represents the expression level of non-cancerous renal tissues (equivalent to 1.0). The Mann–Whitney U test was used for comparison. (**B**) Receiver operating characteristic curve analysis of HSP70 expression level for discriminating patients with RCC from the non-cancerous group. AUC: area under the curve. *p*-value < 0.05 was considered statistically significant.

The fold change of HSP70 in RCC tissues was compared to paired non-cancerous renal tissues in each patient. HSP70 expression was upregulated in RCC tissues from almost all patients (median = 6.71, IQR = 2.57–21.97) (*p* < 0.001) (Figure 2A). ROC curve analysis showed an area under the curve of 0.902 (sensitivity = 84%, specificity = 60.2%, *p* = 0.001) (Figure 2B).

### 3.3. HSP70 Gene Expression Signature and the Clinicopathological Features of RCC

**Figure 3:**
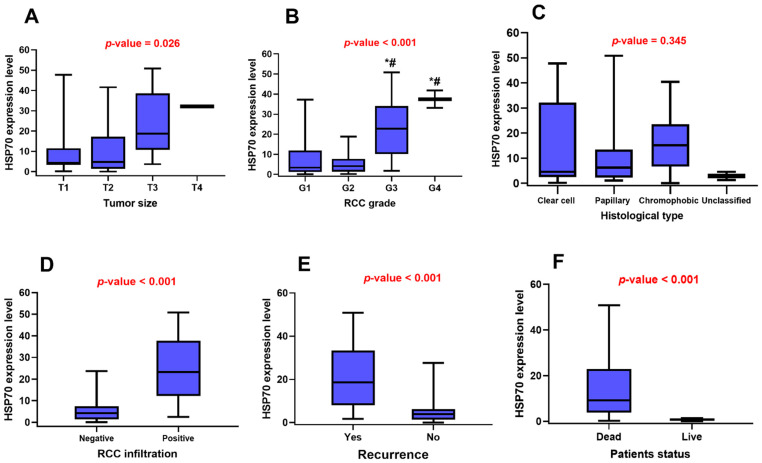
Association of *HSP70* gene expression with clinicopathological characteristics. (**A**) Tumor size, (**B**) Pathological grade, (**C**) Histological subtype, (**D**) Capsular infiltration, (**E**) Recurrence, and (**F**) Mortality. Mann–Whitney U and Kruskal–Wallis tests were used for comparison. *p*-value < 0.05 was considered statistically significant. * Significance versus G1, ^#^ Significance versus G2.

HSP70 signature was higher in tumor samples with large sizes (*p* < 0.026), advanced pathological grade (*p* < 0.001), capsular infiltration (*p* < 0.001), RCC recurrence (*p* < 0.001), and mortality (*p* < 0.001) (Figure 3). Moreover, post-hoc analysis was performed to detect the differences among groups in each category. HSP70 expression was higher in G3 and G4 RCC when either was compared with G1 and G2 tumors (Figure 3B). However, there were no significant differences among the categories of tumor size and histological subtype (Figure 3A,C).

**Figure 4:**
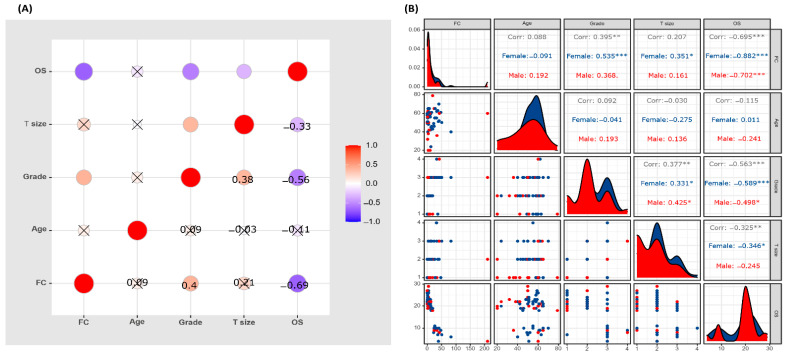
Correlation matrix of *HSP70* expression levels and clinicopathological characteristics of renal cell carcinoma (RCC). Correlation plot showing the relation between *HSP70* expression and the clinicopathological characteristics in RCC patients. OS: overall survival, T: tumor size, FC: fold change. Pearson correlation analysis was employed. (**A**) Overall analysis. (**B**) Stratification by sex. Male in red and female in blue. * *p* < 0.05, ** *p* < 0.01, *** *p* < 0.001.

As depicted in the correlation matrix (Figure 4), we found a positive correlation between *HSP70* gene expression and pathological grade (*r* = 0.395, *p* = 0.001) and a negative correlation with survival time (*r* = −0.695, *p* < 0.001). However, stratification analysis by sex revealed disparities. The correlation coefficients for the pathological grade were *r* = 0.535 (*p* < 0.001) in females and were not significant in males (*r* = 0.368, *p* = 0.09). Similarly, the gene expression level was directly correlated to tumor size in females (*r* = −0.351, *p* = 0.026) but not correlated in male patients (*r* = 0.161, *p* = 0.47). The fold change was negatively correlated with overall survival (OS) in females (*r* = −0.882, *p* < 0.001) and not in males (*r* = −0.702, *p* < 0.001).

### 3.4. Survival Analysis

**Figure 5:**
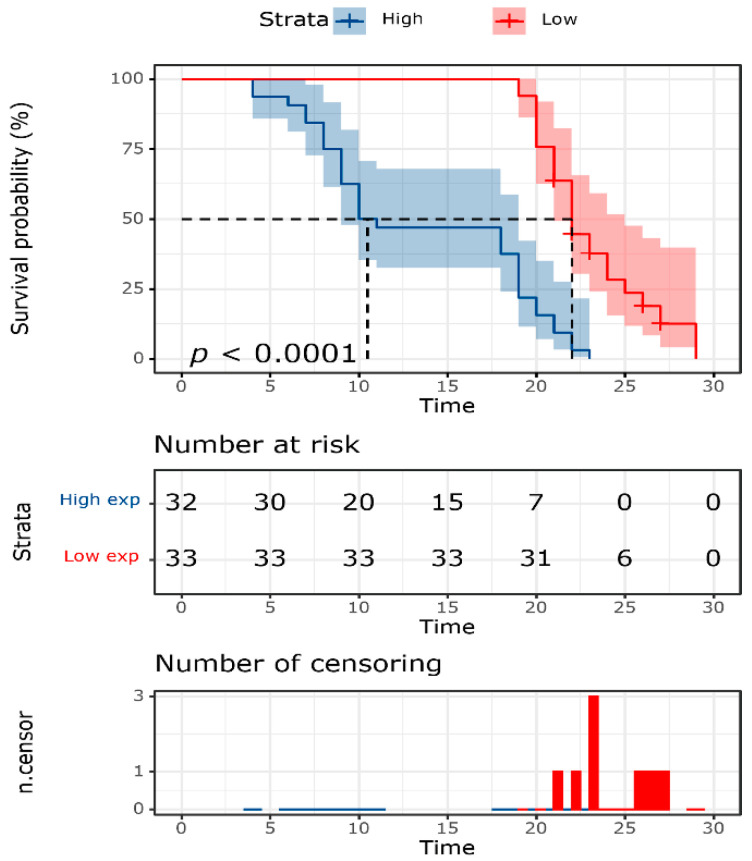
Kaplan–Meier curve of overall survival of patients with renal cell carcinoma stratified by the median value of HSP70 expression level (*p* < 0.05).

The average OS of RCC patients was 18.45 ± 0.84 months. Patients with high *HSP70* expression had reduced OS compared to their counterparts (13.6 ± 1.09 months vs. 23.1 ± 0.6 months, *p* < 0.001) (Figure 5).

### 3.5. In-Silico Analysis

#### 3.5.1. HSP70 Structural and Functional Analysis

**Figure 6:**
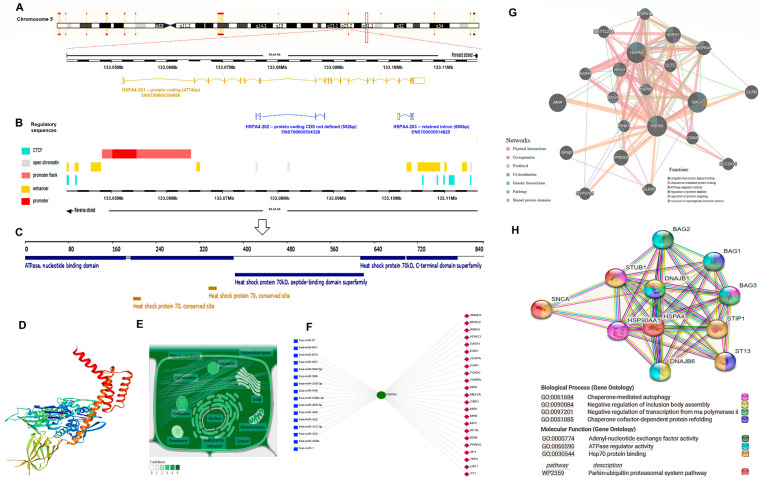
Structural and functional analysis of HSP70 (gene name: *HSPA4*). (**A**) The chromosomal location of the studied gene and the related transcripts. (**B**) The *cis* (e.g., promoter and enhancer sequences) and *trans* (e.g., CCCTC-binding factor; CTCF) acting regulatory factors. (**C**) The 840 amino acid HSP70-related protein product with the different domains and conserved sites. (**D**) The predicted 3D structure of HSP70 generated by “SWISS-modelling”. (**E**) The subcellular localization of HSP70. The color degree is related to protein abundance. (**F**) The transcriptional factor (TF)-miRNA coregulatory interactions include 23 TFs (red diamonds) interacting with the specified gene and 16 microRNAs (blue squares) targeting *HSP70* generated by “Network Analyst 3.0”, filtered by the type of tissue (kidney). (**G**) The “gene- gene interaction network” generated in GeneMania using homo sapiens *HSPA4* as a driver gene. (**H**) The protein-protein interactions network with the top biological processes, molecular functions, and pathways created by the “STRING database”. Each interacting node with the hub HSP70 represents the top 10 predicted functional protein partners with a high level of confidence. The average node degree was 13.8, the average local clustering coefficient was 0.82, and the PPI enrichment *p*-value was <1.0 × 10^−16^.

The *HSP70* gene (*HSPA4*; Gene ID: 3308, ENSG00000170606) maps to the long arm of the human chromosome 5 at the locus 5q31.1, spanning 54,437 bases (133,052,013–133,106,449) on the forward strand according to the GRCh38.p14 primary assembly (Figure 6A). This gene has 19 exons and is transcribed into three different transcripts. The first HSPA4-201 (ENST00000304858.7) transcript is 4774bp protein coding, while the HSPA4-202 (ENST00000504328.1; 552 bp) and HSPA4-203 (ENST00000514825.1; 690 bp) are non-coding transcripts generated by alternative splicing with undefined sequences and due to intron retention, respectively (Figure 6A). Some of the *cis-* and *trans*-acting regulatory sequences and factors that regulate the transcription process are depicted in Figure 6B. 

The encoded protein is 840 amino acids in length (94331 Da) and consists of some essential regions: the amino terminus is the “ATPase domain”, and the carboxyl terminus includes the “substrate-binding region” and the “peptide-binding domain” (Figure 6C). These structural domains assist in several protein functions, including chaperone-mediated protein complex assembly, protein insertion into the mitochondrial outer membrane, and the folding process that involves repeated cycles of the substrate and ATP binding/release as Hsp70 activity is ATP-dependent.

Using ProMod3 v3.2.1 [36], which depends on the “SWISS-MODEL template library” (SMTL version 29 November 2022, PDB release 18 November 2022) to search for related evolutionary structures matching the “HSP74_HUMAN P34932 Heat shock 70 kDa protein 4” sequence for 3D protein structure modeling yielded the result illustrated in Figure 6D. The protein can be found in different cellular compartments, in particular the cytosol and extracellular exosomes (Figure 6E).

As transcriptional/post-transcriptional gene regulations play vital roles in many biological functions and cellular processes, the “visual analytics platform for comprehensive gene expression profiling, NetworkAnalyst 3.0” [37] was applied to identify the enriched transcriptional factors (TFs) and microRNAs (miRNAs) that have a potential impact on HSP70 (Figure 6F). Most of these TFs and miRNAs were implicated in cancers, including RCC [38,39,40].

The predicted gene coexpression network for the hub gene using the default setting of the GeneMania database [41] is illustrated in Figure 6G. The type of gene-gene relationships includes “coexpression, colocalization, genetic interaction, shared protein domain(s), and physical protein interactions”- based on evidence relationships [42].

Among the predicted functional protein partners interacting with HSP70 with high level of confidence are (1) the “DnaJ homolog subfamily B member 1 (DNAJB1)” with confidence level = 0.999; stimulating ATP hydrolysis, ATPase activity, and the folding of unfolded proteins mediated by HSPA1A/B (in vitro), (2) the molecular chaperone “HSP 90-α class A member 1 (HSP90AA1)” with confidence level = 0.989; promoting the maturation/structural maintenance and proper regulation of specific proteins implicated in cell cycle control/signal transduction, (3) the co-chaperone “BAG family molecular chaperone regulators (BAG1,2,3)” with confidence level (= 0.991); acting as “nucleotide-exchange factors” promoting ADP release from HSP70 with subsequent substrate protein release and having anti-apoptotic activity, (4) the “stress-induced-phosphoproteins (STIP)” with confidence level (=0.989); acting as a co-chaperones for HSP90AA1, and (5) the “E3 ubiquitin-protein ligase CHIP (STUB1)” with confidence level (=0.987); an E3 ubiquitin-protein ligase which targets the misfolded chaperone substrates towards proteasomal degradation and modulates HSP70 activity (Figure 6H) (data source: https://string-db.org) (last accessed 1 December 2022).

#### 3.5.2. Analysis of the GTEx Project and TCGA Tissues for the HSP70 Expression in RCC Subtypes

**Figure 7:**
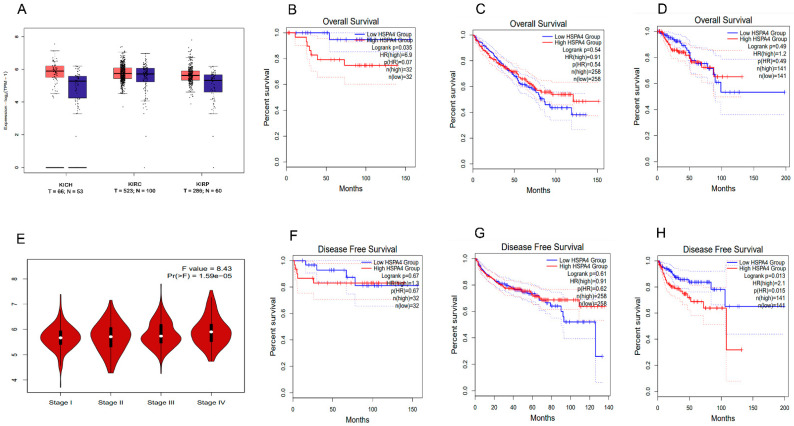
In silico GTEx project and TCGA *HSP70* expression analysis (**A**) differential expression of *HSP70* in three subtypes of renal cell carcinoma (RCC) tumors (T) compared to normal tissues (N) in the GEPIA database. (**B**–**D**) Survival analysis of patients with different RCC subtypes: kidney chromophobe (KICH), kidney renal clear cell carcinoma (KIRC), and kidney renal papillary carcinoma (KIRP)) with high or low *HSP70* expression. (**E**) Association of tumor stage with *HSP70* expression in RCC patients from the GEPIA database. (**F**–**H**) Disease-free survival analysis for patients with high or low *HSP70* expression in different cancer subtypes.

To better understand the potential involvement of HSP70 in carcinogenesis and to validate our results, several bioinformatics databases were screened to quantify HSP70 relative expression in three distinct RCC subtypes. Using the GEPIA database, the *HSP70* expression was evaluated in tumor versus normal tissues from the GTEx project and TCGA samples. The available bioinformatic data comprised 523 KIRC samples, 286 KIRP samples, and 66 KICH samples. Upregulation of HSP70 was confirmed in KICH, KIRC, and KIRP (Figure 7A). The predictive value of HSP70 across the RCC subtypes was next analyzed by comparing patient OS and disease-free survival (DFS) according to HSP70 expression levels. Intriguingly, we discovered that high HSP70 expression was a predictor of poor OS among individuals diagnosed with KIRP (Figure 7D). In contrast, in exploring patients with the KICH and KIRC subtypes, HSP70 expression was not found to be a significant factor in determining patient prognosis (Figure 7B,C). As shown in Figure 7H, patients of the KIRP subtype whose *HSP70* expression was upregulated had a shorter DFS than those of the KICH and KIRC subtypes (Figure 7F,G). Using the GEPIA database, we found a significant association between *HSP70* expression and different RCC stages that support the clinical importance of HSP70 in RCC (Figure 7E).

#### 3.5.3. HSP70 as a Potential Prospective DNA Variation Biomarker in RCC

**Figure 8:**
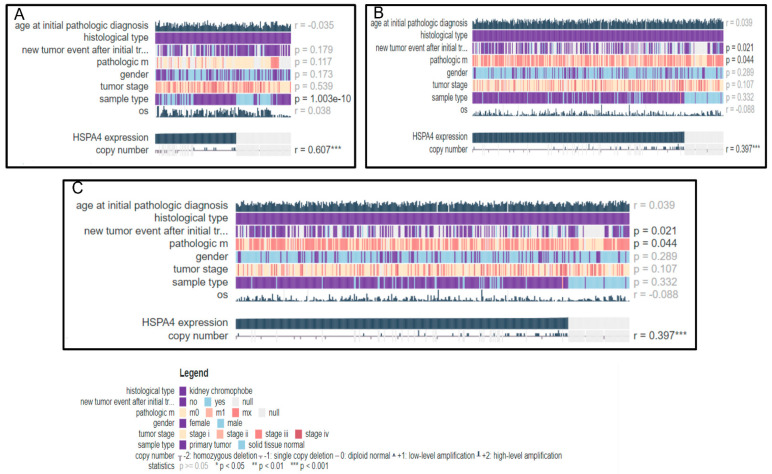
Observation of the MEXPRESS database for HSP70 in renal cell carcinoma (RCC). (**A**) Kidney chromophobe (KICH) subtype, (**B**) Kidney renal clear cell (KIRC) subtype, and (**C**) Kidney renal papillary carcinoma (KIRP) subtype. * *p* < 0.05, ** *p* < 0.01, *** *p* < 0.001.

The MEXPRESS database linked the *HSP70* (*HSPA4*) gene to the age of initiation, recurrence, metastasis, tumor stage, sample type, and OS in the three distinct RCC subtypes. The default MEXPRESS plots from different RCC sample subtypes (KICH (*n* = 149), KIRC (*n* = 980), and KIRP (*n* = 380)) are shown in Figure 8A–C, with the samples sorted based on the *HSP70* expression value. The Pearson correlation coefficients varied from 0.035 to 0.60, and the *p*-value for the comparison of expression with new tumor occurrence and pathological metastasis was 0.021 and 0.04 in KIRC and KIRP, respectively, using the Wilcoxon rank test.

#### 3.5.4. cBioPortal Database Genomic and Target Pathway Analysis

**Figure 9:**
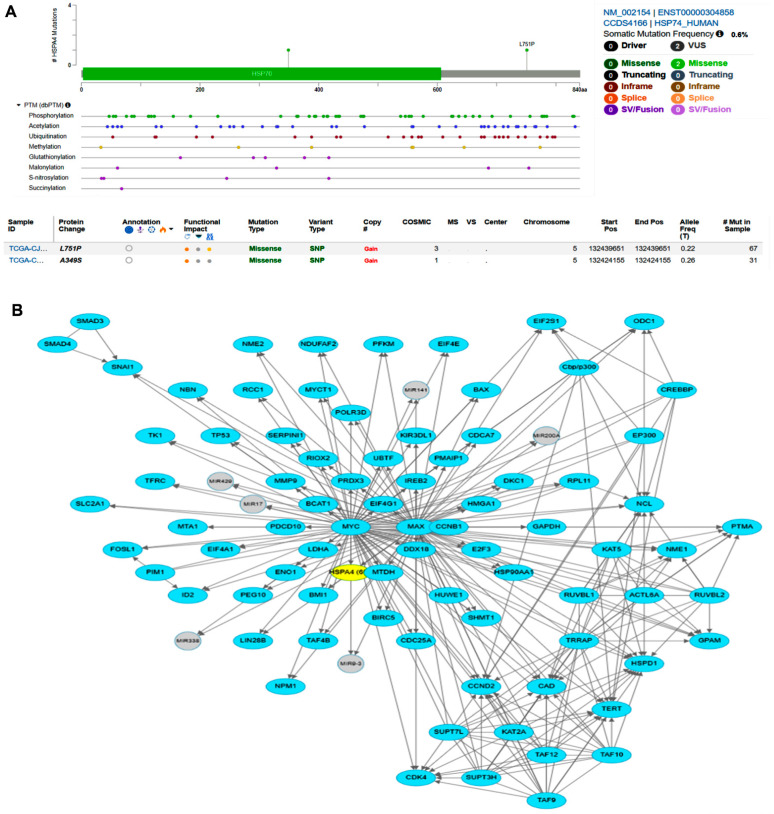
cBioPortal database genomic and network analysis of HSP70 in renal cell carcinoma (RCC). (**A**) Missense mutations that could impact the *HSP70* expression level in RCC. (**B**) *HSP70* is one of the validated targets for C-MYC transcriptional activation.

We screened the genetic variation of *HSP70* in RCC from the cBioPortal database. Results showed that the *HSP70* gene has two hotspots of missense mutations that can alter the HSP70 expression level (Figure 9A). By accessing the pathway network analysis in the cBioPortal database, *HSP70* was found as one of the targets that are involved in C-MYC transcriptional activation and so might be involved in RCC cancer development (Figure 9B).

## 4. Discussion

Despite advances in diagnosis and treatment, RCC is usually diagnosed incidentally, and the 5-year survival rate depends mainly on the stage at diagnosis, with only 12% for metastatic disease [4]. A better understanding of the molecular pathogenesis of RCC is necessary for provisioning earlier diagnostic and improved therapeutic interventions. 

RCC is more common in males; the peak incidence occurs between 60 and 70 years of age, and KIRC is the most common subtype [1]. The epidemiology of RCC varies among different countries and geographic regions. The incidence and mortality are lower in Northern Africa-Middle East, and the disease occurs at an earlier age (in the fifties) compared to North America and Europe [1]. Egypt has the highest mortality rates for RCC in Northern Africa [1]. However, limited data is available on the precise epidemiology of RCC in Egypt [43]. More than half of the RCC patients in our study were in the 21–40-year age group (58.5%). Females represented 66.2% of the study population. KIRC was the most common histological subtype (49.2%). Most patients involved in the study (89.2%) did not survive, and a little more than half did not survive beyond 20 months of diagnosis. Age group and gender in our study population show variation from global findings, which may be explained by the high prevalence of the risk factors (e.g., lifestyle factors, high incidence of associated comorbidities, high parity rate, uncontrolled use of analgesics, etc.) in this region which predispose to increased risk and earlier onset of RCC [1].

HSP70 is a molecular chaperone protein with a multifunctional role in cancer [20,36]. In this study, we investigated the expression levels of *HSP70* in RCC as compared to non-cancerous kidney tissue, and we found that there was a > 6.5-fold increase in expression in cancerous tissue (*p* < 0.001). This is consistent with the finding for *HSP70* expression levels in other cancers, including breast cancer [37,38], gastric cancer [39], cervical carcinoma [40], and bladder urothelial carcinoma [20,41]. This is also consistent with the work of Singh & Suri [22] on RCC, who demonstrated that the knockdown of the *HSP70* gene resulted in the reduction of cellular proliferation and cancer aggressiveness and that of Dall’Oglio et al. in clear-cell type RCC who found high expression level of *HSP70* with metastatic RCC than in low-and-moderate grade cancer [42]. The role of HSP70 in cancer could be due to its implication in the immunogenic cancer reaction [12,13,23]. However, our findings contradict those of Ramp et al., who found reduced expression of *HSP70* in RCC [24].

We further correlated the expression levels of *HSP70* with the clinical and pathological features of RCC. Expression was increased with increased size and advanced grade of the tumor, as well as capsule infiltration, suggesting the role of HSP70 in disease progression. Comparable findings were reported in cervical, bladder cell urothelial, and esophageal squamous cell carcinomas [41,44,45]. Moreover, higher circulating *HSP70* levels were an indicator of tumor progression in acute leukemia [46]. Nevertheless, our findings are in contrast with those of Ramp et al. [24], where *HSP70* expression was of no role in RCC progression. 

We have also demonstrated that HSP70 expression was correlated with RCC recurrence and reduced patient OS and DFS, indicating a prognostic role for HSP70 in RCC, where elevated HSP70 expression may be related to poor prognosis. For RCC, our findings are in line with those of Singh and Suri [22] as well as Dall’Oglio et al. [42]. However, Santarosa et al. reported low HSP70 expression in RCC patients with RCC relapse, considering HSP70 a favorable prognostic factor [21]. RCC OS may vary among countries, not only depending on genetic background, but based on the varying behavioral or environmental variables and comorbidities such as smoking, overweight, elevated blood pressure, and chronic kidney disease, all of which have been found to be risk factors for RCC [1]. Therefore, many possible selection biases could be the cause of these conflicting results.

Numerous studies have highlighted the role of HSP70 in the resistance of several types of cancer to chemotherapy and radiotherapy via various molecular pathways [47]. This supports the high-expression levels of HSP70 displayed in our study and may explain why RCCs are believed to be chemotherapy- and radiotherapy-resistant tumors [5].

The bioinformatic analysis performed in this study has revealed HSP70 upregulation in KICH, KIRC, and KIRP (Figure 7A). Moreover, the high HSP70 expression was a predictor of poor OS in KIRP (*n* = 286) (Figure 7D) but was not a significant factor in OS for the KICH (*n* = 66) and KIRC (*n* = 523) subtypes (Figure 7B,C). Upregulation of HSP70 in KIRP as an indicator of poor OS is consistent with our gene expression data but taking into consideration the limited number of samples available in GTEx and TCGA, it could not be inferred that HSP70 expression is of no value in predicting survival in the other RCC histological subtypes. 

This study comprised a small number of RCC patients from a single ethnicity, so replicating this study with a larger number of patients, increasing the sample size for each histological subtype and in different ethnicities could help in better understanding the HSP70 expression repertoire and its association with OS in RCC.

In this study, assessment of the expression level of circulating HSP70 in RCC patients was not possible since our samples were archival; circulating HSP70 could prove to be a possible non-invasive biomarker of the disease. We also excluded patients receiving radiotherapy and chemotherapy prior to surgical resection of the tumor; assessing the effect of radiotherapy and chemotherapy on both circulating and tissue HSP70 expression in RCC could shed light on its possible role in cancer therapy resistance. Regarding the varying correlations between HSP70 expression and the clinicopathological features of different subtypes of RCC by in silico-analysis, it could be of value to replicate this study through stratifying by subtype with increasing sample size in each subtype.

## 5. Conclusions

HSP70 upregulation in RCC tissue is associated with larger tumor size, more advanced grade, capsular infiltration, increased RCC recurrence, and reduced survival.

## Figures and Tables

**Table 1:** Clinicopathological characteristics of renal cell carcinoma patients (*n* = 65).

Characteristics	Levels	Number	Percentage
**Age, years**	≤20	7	10.8
	21–40	38	58.5
	41–60	20	30.7
**Sex**	Male	22	33.8
	Female	43	66.2
**Location**	Right side	44	67.7
	Left side	21	32.3
**Tumor size**	T1	19	29.3
	T2	33	50.7
	T3	12	18.5
	T4	1	1.5
**Pathological grade**	G1	10	15.4
	G2	30	46.1
	G3	23	35.4
	G4	2	3.1
**Histological type**	KIRC	32	49.2
	KIRP	19	29.2
	KICH	12	18.5
	Unclassified	2	3.1
**Capsular infiltration**	Absent	39	60
	Present	26	40
**Recurrence**	Negative	34	52.3
	Positive	31	47.7
**Mortality**	Alive	7	10.8
	Died	58	89.2
**Overall survival, months**	≤20	35	53.8
	>20	30	46.2

Data are presented as numbers (percentages). KICH: kidney chromophobe; KIRC: kidney renal clear cell carcinoma; KIRP: kidney renal papillary carcinoma.

## Data Availability

All generated data in this study are included in the article.
